# 1-Methylcyclopropene Modulates Physiological, Biochemical, and Antioxidant Responses of Rice to Different Salt Stress Levels

**DOI:** 10.3389/fpls.2019.00124

**Published:** 2019-02-21

**Authors:** Sajid Hussain, Zhigang Bai, Jie Huang, Xiaochuang Cao, Lianfeng Zhu, Chunquan Zhu, Maqsood Ahmed Khaskheli, Chu Zhong, Qianyu Jin, Junhua Zhang

**Affiliations:** State Key Laboratory of Rice Biology, China National Rice Research Institute, Hangzhou, China

**Keywords:** rice cultivar, 1-methylcyclopropene, ethylene, sodium chloride, antioxidants, photosynthesis

## Abstract

Salt stress in soil is a critical constraint that affects the production of rice. Salt stress hinders plant growth through osmotic stress, ionic stress, and a hormonal imbalance (especially ethylene), therefore, thoughtful efforts are needed to devise salt tolerance management strategies. 1-Methylcyclopropene (1-MCP) is an ethylene action inhibitor, which could significantly reduce ethylene production in crops and fruits. However, 1-MCPs response to the physiological, biochemical and antioxidant features of rice under salt stress, are not clear. The present study analyzed whether 1-MCP could modulate salt tolerance for different rice cultivars. Pot culture experiments were conducted in a greenhouse in 2016–2017. Two rice cultivars, Nipponbare (NPBA) and Liangyoupeijiu (LYP9) were used in this trial. The salt stress included four salt levels, 0 g NaCl/kg dry soil (control, CK), 1.5 g NaCl/ kg dry soil (Low Salt stress, LS), 4.5 g NaCl/kg dry soil (Medium Salt stress, MS), and 7.5 g NaCl/kg dry soil (Heavy Salt stress, HS). Two 1-MCP levels, 0 g (CT) and 0.04 g/pot (1-MCP) were applied at the rice booting stage in 2016 and 2017. The results showed that applying 1-MCP significantly reduced ethylene production in rice spikelets from LYP9 and NPBA by 40.2 and 23.9% (CK), 44.3 and 28.6% (LS), 28 and 25.9% (MS), respectively. Rice seedlings for NPBA died under the HS level, while application of 1-MCP reduced the ethylene production in spikelets for LYP9 by 27.4% compared with those that received no 1-MCP treatment. Applying 1-MCP improved the photosynthesis rate and SPAD value in rice leaves for both cultivars. 1-MCP enhanced the superoxide dismutase production, protein synthesis, chlorophyll contents (chl *a*, *b*, carotenoids), and decreased malondialdehyde, H_2_O_2_, and proline accumulation in rice leaves. Application of 1-MCP also modulated the aboveground biomass, and grain yield for LYP9 and NPBA by 19.4 and 15.1% (CK), 30.3 and 24% (LS), 26.4 and 55.4% (MS), respectively, and 34.5% (HS) for LYP9 compared with those that received no 1-MCP treatment. However, LYP9 displayed a better tolerance than NPBA. The results revealed that 1-MCP could be employed to modulate physiology, biochemical, and antioxidant activities in rice plants, at different levels of salt stress, as a salt stress remedy.

## Graphical Abstract

1-Methylcyclopropene modulates physiological, biochemical, and antioxidant responses to different salt stress levels in rice.

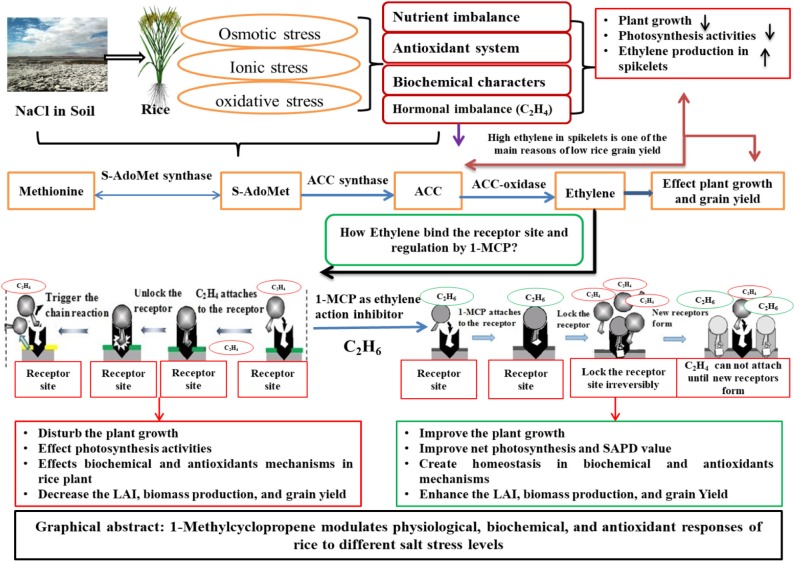

## Introduction

Due to a high and increasing annual population rate, crop production needs to be increased by 87% by 2050 (Kromdijk and Long, 2016). However, abiotic stresses such as salinity, high temperatures and drought are main factors that reduce crop growth and yield, in large areas of the world (Pareek et al., 2010; Mantri et al., 2012). Globally, abiotic stress, such as salinity is a crucial hurdle preventing the increase of crop production in growing areas (Munns, 2011; Zhang et al., 2017). Globally, approximately 20% of irrigated agricultural land is under salt stress, as reported by the United Nations Environment Program (UNEP) (Nellemann et al., 2009; Ruan et al., 2010). The area of salt-affected soil is increasing due to global warming, high temperatures and rising sea levels (Rabbani et al., 2013; Islam et al., 2015a). Excessive salt stress can induce several adverse effects in plants, such as osmotic, ionic, and oxidative stress, hormonal imbalances (ethylene production in crops), through the accumulation of Na^+^ and Cl^-^ in plant tissues, and the over-production of reactive oxygen species (ROS) (Miller et al., 2010; Sajid et al., 2018).

Rice is a salt-sensitive crop (Roy et al., 2014; Parihar et al., 2015). Areas affected by salinity are expanding, becoming a critical global issue that obstructs the sustainability of rice production (Hairmansis et al., 2017). Osmotic stress, due to salt-induced stomatal closure, increases leaf temperature and inhibits shoot growth (Rajendran et al., 2009; Roy et al., 2014). Therefore, through osmotic stress, salt stress inhibits plant growth and directs the senescence of older leaves in plants (Islam et al., 2017a,b). Generally, salt stress has numerous inhibitory effects on rice plants, including reduced leaf emergence, shoot cell enlargement, photosynthesis rates, chlorophyll contents, and water use efficiency (Amirjani, 2010). Salt stress also significantly influences the leaf area index and dry matter production in rice plants (Amirjani, 2010; Sajid et al., 2018). Salt stress disturbs the balance between the scavenging and production of ROS in plants. This disequilibrium can cause significant damage to cell structures through a sharp escalation of ROS in intracellular tissues (Sharma et al., 2012).

Reactive oxygen species are located in various cellular components such as mitochondria, peroxisomes, and chloroplasts (Del Rio et al., 2006; Navrot et al., 2007). As a result, they can harm the chloroplast pigments, proteins, membrane lipids, enzymes, and nucleic acids (Del Rio et al., 2006). The balance between the production and scavenging of ROS is critical for the survival of plants in unfavorable environments. The glyoxalase systems and antioxidant defense system is intermixed. Therefore, the proficiency of these systems needs to be interrelated to improve tolerance levels of abiotic stressors in numerous crops (Mustafiz et al., 2011).

Numerous plant types can protect themselves from osmotic and ionic stress through biosynthesizing friendly solutes, such as trehalose and proline, stabilizing membranes, and by sustaining water relations, enzymes, and complexes of proteins (Iqbal et al., 2015; Reddy et al., 2015). Protection mechanisms against salt stress persuaded-ROS in plants, are convincingly linked with the conservation of cellular redox equilibrium, which is mostly comprised of a list of antioxidants with an enzymatic nature, such as catalase (CAT), malondialdehyde (MDA), superoxide dismutase (SOD), glutathione S-transferase (GST), and glutathione peroxidase (GPX), along with antioxidants with a non-enzymatic nature, such as ascorbic acid (AsA) and glutathione (GSH) (Shao et al., 2007; Miller et al., 2010).

Ethylene is a key signaling hormone, but its role in rice plants that are under salt stress, is quite complex (Tao et al., 2015). A high accumulation of Na^+^ in rice plants that are under salt stress, increases ethylene production and various enzymatic activities in rice (Zhao and Schaller, 2004). High ethylene release also increases the sensitivity to salt stress (Yang et al., 2014), which leads to plant death. Some studies reported that overexpression of ACSs increased sensitivity to salt and increased ethylene production in plants (Li et al., 2014; Tao et al., 2015). The enzymatic mechanisms interrelated to the assembly of starch in rice grains, were also affected by high ethylene production at the rice grain filling stage, which then reduced the rice grain yield (Zhu et al., 2011; Zhang et al., 2014). In genetic manipulation, the inhibition of ethylene production, through chemical means, is also an alternative approach. The use of ethylene action inhibitors, such as ethephon, 1-methylcyclopropene (1-MCP), and amino-ethoxy vinyl glycine (AVG) are valuable for crops to retain plant yields (Zhang et al., 2009).

Among the ethylene action inhibitors, 1-MCP is an efficient chemical means, which has shown an inhibition to the ethylene-inducible effects on various plant species (Yang et al., 2007). 1-MCP is small and has a low molecular weight, and α-cyclodextrin (α-CD) complex can capture gaseous 1-MCP, which confirms that it is an environmentally friendly chemical (Trotta et al., 2011). 1-MCP binds ethylene receptor sites irreversibly; therefore, subsequent signal translocation and transformation reactions, related to ethylene, are not provoked (Blankenship and Dole, 2003). It was also reported that binding ethylene receptors with 1-MCP reduced protein synthesis and enzymes which were activated by ethylene production under stress (Su and Finlayson, 2012). Modern research has shown that the 1-MCP application improved dry matter segregation, the photosynthesis rate, and chlorophyll content, and also promoted carbon assimilation in rice plants (Wang et al., 2012; Zhang et al., 2014, 2015).

This study was conducted, keeping all the information mentioned above in mind, to prove the hypothesis that higher ethylene production in rice plants, especially in spikelets, is one of the main reasons for lower rice physiological development and grain yield under salt stress conditions. The physiological, biochemical, and antioxidant response to salt stress of different rice cultivars, and the regulation of 1-MCP have not been studied before.

The primary objective of this study was to evaluate the responses of 1-MCP on physiological, biochemical, and antioxidant characteristics in rice under salt stress. The list of physiological traits in rice included net photosynthesis rate (P_n_) and chlorophyll contents (SPAD values) at the rice full heading stage. The antioxidants attribute involved quantifying SOD, MDA, H_2_O_2_, biochemical features, i.e., Chlorophyll contents (chlorophyll a, b, carotenoids, and total chlorophyll), proline, and the soluble protein concentration in rice flag leaves. Along with the parameters mentioned above, the aboveground biomass and grain yield for rice plants were also measured at maturity. These results will be useful for evaluating the relative abilities of rice cultivars being cultivated in the region and will establish the potential commercial use of 1-MCP against salt stress.

## Materials and Methods

### Growth Conditions and Experiment Design

The pot culture experiments were conducted in the greenhouse of the China National Rice Research Institute (31°4′49″ N, 119°56′11″ E), Zhejiang Province, China, in 2016 and 2017. Two rice cultivars, Nipponbare (NPBA, Japonica) and Liangyoupeijiu (LYP9, Indica) were used in this trial. The soil texture used in this experiment was loam clay. Soil (30 kg per pot) was air-dried, ground, and sieved, and filled into pots (45 × 30 cm). Six rice seedlings (30-days old) were then transplanted into each pot.

A chemical reagent, sodium chloride (NaCl), was used as a salt stress agent in the soil. Four levels of NaCl were used, including 0 g NaCl/ kg dry soil (control, CK), 1.5 g NaCl/ kg dry soil (Low Salt stress, LS), 4.5 g NaCl/ kg dry soil (Medium Salt stress, MS), and 7.5 g NaCl/ kg dry soil (Heavy Salt stress, HS), respectively. The salt was homogenized with air dried soil before the seedlings were transplanted. The electrical conductivity (EC) values for CK in 2016 and 2017 were 0.0864 dS m^-1^ and 0.154 dS m^-1^, for LS were 1.089 dS m^-1^ and 1.16 dS m^-1^, for MS were 3.2 dS m^-1^ and 3.27 dS m^-1^, and for HS were 4.64 dS m^-1^ and 4.71 dS m^-1^, respectively. All pots were divided into two parts, 1-MCP was applied in half of the pots, while the other half was used as the control. The 1-MCP was applied at the rice mid-booting phase with an amount of 0.04 g per pot during 2016 and 2017. Each treatment was replicated three times. The time frame of the crop lifecycle, from sowing to maturity, is given in Table 1.

**Table 1:** Crop data in 2016 and 2017.

Year	Cultivars	Sowing dates	Plant density	Transplanting	Maximum tillering	Full heading	Maturity
		mm/dd	Seedlings/pots	mm/dd	mm/dd	mm/dd	mm/dd
2016	NPBA	05/25	6 seedlings	06/23	07/28	08/16	10/10
	LYP9	05/25	6 seedlings	06/23	08/04	08/23	10/20
2017	NPBA	05/24	6 seedlings	06/22	07/31	08/19	10/13
	LYP9	05/24	6 seedlings	06/22	08/07	08/30	10/17


*NPBA, Nipponbare; LYP9, Laiangyoupeijiu.*

Nipponbare could not survive at the HS level, and all plants died within 15 days of exposure to salt stress after transplanting. Thus, the NPBA results only included three salt stress levels (CK, LS, and MS), and the LYP9 results included all salt stress levels (CK, LS, MS, and HS). In 2017, similar responses were observed in NPBA and LYP9 at HS level. The results for LYP9 and NPBA in 2017 only included the three salt stress levels.

The experimental soil consisted of loamy clay with a 1.12 g cm^-3^ bulk density, containing 4.7% organic matter with pH 5.95, and the represented EC was 0.12 dS m^-1^. Nitrogen was applied in the form of urea (N 46%), phosphorous was used as superphosphate (P_2_O_5_ 12%), and potassium was used as potassium sulfate (K_2_O 54%). The urea was used at the rate of 4.02 g and 5.24 g per pot in three splits; basal dose (50%), top dressing at rice tillering stage (30%), and top dressing at rice booting stage (20%), respectively. The superphosphate (6.93 g per pot and 9.04 g per pot) was applied as a basal dose. Potassium sulfate (3.08 g per pot and 4.02 g per pot) was added in two splits, basally (50%), and a top dressing at the rice booting stage (50%) during 2016 and 2017, respectively.

### 1-Methylcyclopropene Application

Gassy 1-MCP was set free in granules (Fresh Doctor, Xianyang XiQin Biological Technology Co. Ltd). 1-MCP gas was released by dissolving the powdered formulation into the water. The 1-MCP was applied at the mid-booting stage at 0.04 g per pot during 2016 and 2017. A plastic sheet was used to cover the pots after 1-MCP application to provide a closed environment for the reaction, and pots were uncovered for as long as 24 h (Harper, 2015; Sajid et al., 2018). The irrigated water level was sustained at about 2–4 cm in the pots, while 1-MCP was applied.

### Plant Sampling

Plant samples from different treatments were collected and measured. The plant length, root length, leaf area and the dry matter (DM) at the rice full heading stage were measured. The ethylene of superior spikelets and inferior spikelets were collected with three replicates. Three plant samples were collected for measurement of the aboveground biomass at rice maturity stage. Flag leaves from five rice plants were sampled after 1-MCP application with three replicates, and SOD, MDA, H_2_O_2_, chlorophyll a, b, carotenoids, proline, and soluble protein in rice flag leaves were measured every 10 days.

### Ethylene Extraction

The ethylene production in spikelets was calculated following Beltrano et al. (1994) and Zhang et al. (2014). Thirty spikelets (superior and inferior spikelets) were separated from panicles and placed between two moist papers for 60 min at room temperature (27°C) in the dark, to eliminate the ethylene trauma caused by cutting. The treated spikelets were shifted into a 70 mL glass vial and instantly wrapped with an airtight stopper. The samples were incubated in the dark for 24 h at 27°C. A 1 mL gas sample was withdrawn from the vial using a syringe, and the concentration of ethylene was calculated by using gas chromatography (Agilent 7890A, Agilent Technologies Inc., United States) furnished with an FID (flame ionization detector) and column GS-GASPRO. The temperature was kept at 160°C, and 150°C for the detector, and the injection port was held constant. Nitrogen gas worked as a carrier gas with a 14 mL min^-1^ flow rate. The total 70 ml of the gas volume was used. The concentration of ethylene was calculated with pmol unit g^-1^ (FW) h^-1^.

### Measurement of Proline and Soluble Protein in Flag Leaf

Soluble protein in rice flag leaves were measured according to the methods of Sedmak and Grossberg (1977) and Scopes (1987). The soluble protein solution extraction was absorbed into a tube, and 5 ml of Coomassie brilliant blue G-250 (Bradford assay) staining fluid was added and thoroughly mixed. The tube was then set aside for 20 min. For protein assays, Bovine serum albumin (BSA) was used as a standard. The absorbance was measured chronometrically (UV-2600, Shimadzu, Japan) at 595 nm. Soluble protein was measured using the given formula:

$$\text{Soluble Protein }(\text{mg/g fresh weight})=C\times \frac{\left(\frac{V}{v}\right)}{\left(w\times 1000\right)}$$

Where, *C* is the concentration of samples, *W* (g) is the fresh weight of samples used during measurement, *V* (mL) is the total volume of the extract, and *v* (mL) is the volume of extract added to the reaction.

Proline in flag leaves (about 0.2 g) was extracted with 5 ml of sulfosalicylic acid (3%) in a boiling water bath for 30 min. After that, filtrated extraction (2 ml) was mixed with a ninhydrin reagent (2 ml) and glacial acetic acid (2 ml). This combination of the mixture was put at 100°C into boiling water for 30 min and then put on ice (-4°C) for 20 min before extraction with toluene (4 ml). The chromospheres absorbance in the toluene fraction was calculated colorimetrically at 520 nm using a UV-VIS (Spectrophotometer Shimadzu). The amount of proline was determined by comparing it to a calibration curve prepared with an l-proline solution. The proline was calculated using the following formula (Bates et al., 1973):

$$\text{Proline contents=C}\times V\times \frac{2}{a\times w}$$

Where *C* is the concentration of the sample, *V* is the volume of the extracted solution, *a* is the standard solution, and *w* is the weight of the sample. The unit of proline was used as μM g^-1^ FW.

### Chlorophyll Contents

Chlorophyll contents (Chl *a*, *b*, and carotenoids) were extracted from fresh flag leaves using 25 mL of mixed acetone and alcohol (v:v = 1:1) for 24 h in the darkness at 25°C. The absorbance of the sample was calculated at 663, 645, and 470 nm using a UV-VIS spectrophotometer (UV-2600, Shimadzu, Japan) to estimate the chlorophyll contents according to the methods by Wellburn and Lichtenthaler (1984) and Zhong et al. (2017).

The following formulas were used to calculate the Chl a, b, carotenoids, and total chlorophyll:

Chlorophyll a=12.21A663−2.81A645

Chlorophyll b=20.13A645−5.03A663

$$Carotenoids=\frac{1000A470-3.27Ca-104Cb}{229}$$

Total Chlorophyll contents=Chl a + Chl b + Carotenoids

The unit for chlorophyll contents was used as mg.g^-1^ FW.

### Determination of Antioxidants

#### SOD in Rice Flag Leaves

Frozen flag leaf samples were crushed in liquid nitrogen and mixed with a 6 ml sodium phosphate buffer (pH 7.8). This mixture was centrifuged at 6000 rpm for 10 min at 4°C, and the supernatant was put in storage at -70°C for the measurement of soluble protein, SOD, and MDA. SOD activity was assayed by employing the methodology of Giannopolitis and Ries (1977) and Agarwal and Pandey (2004). The SOD reaction system contained 25 mmol NaH_2_PO_4_ buffer, 13 mmol methionine, 10 μmol/L riboflavin, 100 μmol/l EDTA-Na, 750 μmol/l nitro blue tetrazolium (NBT), and a 0.1 mL enzyme extraction was added to the reaction. Samples were put under light (4000 lx) for 20 min, and the absorbance was calculated chronometrically at 560 nm. The blank replaced the extract with a sodium phosphate buffer and was placed in darkness. SOD action was demarcated as the enzymatic amount that would obstruct a 50% photoreduction of nitro blue tetrazolium (NBT) chloride. The unit of SOD used was μg^-1^FW h^-1^. The SOD activity was calculated using the following equation:

$$SOD\,\;activity\,=(ODc-ODs)\times \frac{v}{\left(0.5\times ODc\times Vs\times m\right)}$$

Which includes *ODc* (*ODc* of control tube), *ODs* (*ODs* of sample tube), *V* (mL) (volume of enzyme extraction), *Vs* (mL) (volume of enzyme extraction added to reaction), and *m* (g) (weight of sample).

#### Malondialdehyde in Rice Flag Leaves

The methods of Velikova et al. (2000) measured the MDA value in flag leaves of rice. A tube consisting of 1 ml extract and 2 ml thiobarbituric acid (TBA, 0.5%) in 0.5% trichloroacetic acid (TCA) was placed in boiling water to react for 15 min. The absorbance was measured chronometrically (UV-2600, UV-VIS Spectrophotometer Shimadzu) at 450, 532, and 600 nm. The quantity of the MDA-TBA complex was determined from the extinction coefficient at 155 mmol L^-1^. The MDA was calculated as μmol/g fresh weight.

C−6.46×(D532−D600)−0.56×D450

Where D450, 532, and 600 are wavelengths, and

MDA=C×4×2.5/(1.55×W)

Where *C* is the concentration at a different wavelength, and *W* is the sample weight.

#### H_2_O_2_ in Flag Leaves

H_2_O_2_ values were calculated using previous methods by Brennan and Frenkel (1977) and Yang et al. (2007). About 0.1 g frozen flag leaves were crushed in liquid nitrogen and mixed with 3 mL of 10 mM 3-amino-1,2,4-triazole. The extract was centrifuged at 8000 rpm for 10 min at 4°C. After centrifugation, 2 mL supernatant extract and 1 mL titanium sulfate (0.1%) in 20% sulfuric acid was mixed in 10 mL-centrifuge tubes. The samples were placed at room temperature for 10 min, the mixture was centrifuged again at 8000 rpm for 10 min at 4°C. The supernatant extract was calculated calorimetrically at 410 nm. A standard curve was obtained using 30% H_2_O_2_ as a standard.

$$H2O2=\frac{C*\left(V/Vs\right)}{W}$$

Where *C* is absorbance, *V* is total volume, *Vs* is extracted volume, and *W* is leaf sample weight. The unit of H_2_O_2_ was used as μmol.g^-1^ FW.

### Physiological Parameters

#### Photosynthesis

The net photosynthesis rate (P_n_) of the flag leaf was recorded at the rice full heading stage after 1-MCP application. The photosynthesis data were recorded using a portable photosynthesis system (LI-6400XT, LI-COR Company, United States) on sunny days between 8:30 am to 11:30 am. In the leaf chamber, the CO_2_ concentration was kept at 400 μmol mol^-1^. The temperature was set at 30°C, and the light intensity was set to 1500 μmol Em^-2^ S^-1^, at the time of recording.

#### SPAD Value

Ten rice plants were selected to measure chlorophyll contents (SPAD values) at the rice full heading stage. The reading was taken at 7 days intervals, from the same plants. SPAD values of the rice flag leaves were measured three times after application of 1-MCP by a SPAD meter (SPAD-502 plus).

### Aboveground Biomass and Grain Yield

Three plant samples were harvested at maturity, to measure the total aboveground biomass. The plants were harvested carefully from the near soil surface and placed in room temperature until samples gained a constant weight. Three plant samples were harvested for calculating the grain yield.

### Quality Assurance

Our laboratory, the State Key Laboratory of Rice Biology in China, is ranked as an international laboratory. The integrity of our laboratory systems is routinely audited and certified by the British standards institution and meet all applicable requirements of the ISO9001 standard. All chemicals were purchased by the Aladdin Industrial Corporation, Shanghai, China and the Sigma-Aldrich Corporation China, and the purity level was 99%.

### Statistical Analysis

This experimental design used was a completely randomized design (CRD). Statistical analysis of the data was compiled using standard analyses of variance (Two-way ANOVA). The comparison of mean values was made using the least significant difference (LSD) at the level of significance (5%) using SPSS Statistics (19.0 software package).

## Results

### 1-MCP Modulated Physiological Characters of Rice Under Salt Stress

#### Net Photosynthesis Rate

**FIGURE 1:**
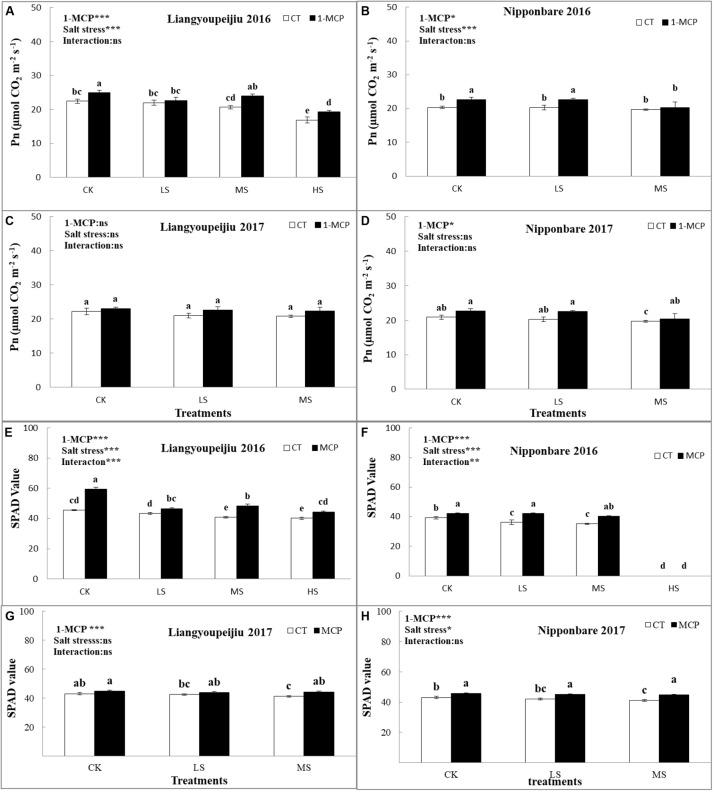
Comparison effects of 1-MCP and salt levels on net photosynthesis rate, P_n_ **(A–D)** and SPAD values **(E–H)** of rice flag leaves in 2016 and 2017. Values are denoted as mean ± SE (P_n_, *n* = 3 and SPAD, *n* = 5). Values followed by different letters are significantly different (*p* ≤ 0.05) according to the LSD test. Where treatments are Control (CK), Low Salt Stress (LS), and Medium Salt Stress (MS), while CT = no 1-MCP treatment and 1-MCP = 1-MCP treatment. Liangyoupeijiu (LYP9) and Nipponbare (NPBA) are rice cultivars. *P*-values of the two-way ANOVAs of 1-MCP, salt stress, and their interaction are indicated: ns, not significant; ^∗^*P* < 0.05; ^∗∗^*P* < 0.01; ^∗∗∗^*P* < 0.001. Bars with the same letter are not significantly different in the LSD test.

Salt stress in soil decreased the P_n_ values in rice flag leaves for both LYP9 and NPBA (Figure 1A–D). In comparison with the CK group, the P_n_ value for LYP9 was 2.3, 8.4, and 25.1% lower in the LS, MS, and HS treatments, respectively. In NPBA, the P_n_ value was 2 and 3.4% higher in LS and MS, compared to CK, respectively. The application of 1-MCP significantly improved the P_n_ value in rice flag leaves under salt stress, compared with no usage of 1-MCP in both rice cultivars (Figure 1A–D). However, in LYP9, the average P_n_ in rice flag leaves was 8.1% (CK), 13.1% (LS), 14.6% (MS), and 14.2% (HS), higher in leaves that received 1-MCP compared to those that received no 1-MCP, respectively (Figure 1A,C). The P_n_ value of LYP9 flag leaves showed a significant difference between increased salt stress treatments (MS and HS).

For NPBA, the P_n_ value of flag leaves were considerably lower with increased salt stress levels. The average P_n_ of rice flag leaves were 12.9% (CK), 19.9% (LS), and 14.3% (MS) higher in leaves that received 1-MCP compared to those that received no 1-MCP (Figure 1B,D). These results revealed that 1-MCP had significant effects on increasing the P_n_ value of rice flag leaves in the CK and LS treatments, but had no significant effects in the MS level. This could be an intrinsic factor of the NPBA cultivar in response to salt stress.

Overall, 2 years of data revealed that the value of P_n_ in flag leaves was higher in LYP9 than in NPBA under salt stress, and that NPBA could not survive at the HS level. A high P_n_ value is a source of high carbohydrate formation. In previous studies, due to salt stress, the production of carbohydrates was reduced (Parida and Das, 2005), which ultimately led to a reduction in rice grain yield. Application of 1-MCP improved the P_n_ value of flag leaves in both NPBA and LYP9, even under salt stress conditions. The results showed that 1-MCP has the potential to assimilate carbohydrates in rice leaves, and that it can improve important physiological parameters in rice plants, such as photosynthesis in adverse environments (Figure 1A–D). These findings confirmed the affinity of 1-MCP to improve assimilation of carbohydrates in rice leaves, by enhancing key parameters related to physiology, such as P_n_ under adverse salt stress.

#### SPAD Value

Chlorophyll contents were represented as SPAD values of the flag leaf in this experiment (Esfahani et al., 2008). The LYP9 and NPBA showed a variation in SPAD values in response to salt stress levels (Figure 1E–H). The average of 2 years of data showed that SPAD values in LYP9 decreased by 1.9 and 5.6% in LS and MS levels, compared with CK, respectively. The SPAD values in rice flag leaves were improved by 7.8% (CK), 4.0% (MS), and 8.8% (HS), respectively after 1-MCP application, compared to no 1-MCP in LYP9 (Figure 1E,G).

Whereas, in the NPBA, SPAD values in rice flag leaves reduced by 2.5 and 6% in LS and MS, respectively, compared with the CK. SPAD values in flag leaves were enhanced by 5.7, 6.7, and 8.5% in the CK, LS, and HS, respectively, with 1-MCP usage, compared to no 1-MCP in NPBA. These outcomes support that 1-MCP has important effects on delaying the senescence of rice leaves. NPBA showed a high sensitivity to the HS level and could not survive HS treatment (Figure 1F,H).

The variation of SPAD values between LYP9 and NPBA flag leaves, may be endorsed by the inherent genetic variabilities of both rice cultivars (Figure 1E–H). By comparing LYP9 and NPBA, LYP9 showed higher SPAD values in flag leaves that received 1-MCP, under all salt stress levels, than those for NPBA. The results suggest that 1-MCP has a significant effect on deferring the senescence of flag leaves. The LYP9 showed susceptibility under the adverse condition of HS treatment.

### 1-MCP Modulated Biochemical Characteristics

#### Ethylene Production

**FIGURE 2:**
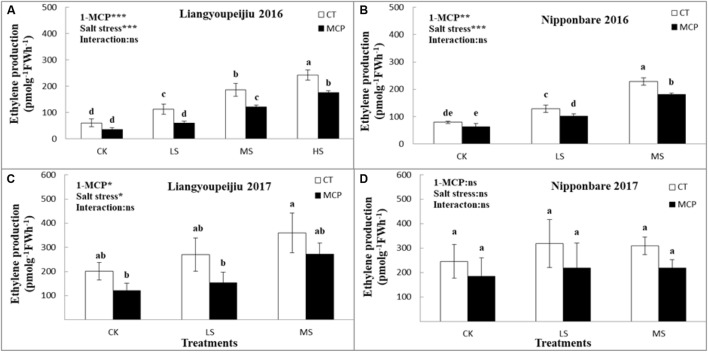
Comparison of effects of 1-MCP and salt levels on ethylene production in spikelets of rice **(A–D)** in 2016 and 2017. Values are denoted as mean ± SE (*n* = 6). Values followed by different letters are significantly different (*p* ≤ 0.05) according to the LSD test. Where treatments are Control (CK), Low Salt Stress (LS), and Medium Salt Stress (MS), while CT = no 1-MCP treatment and 1-MCP = 1-MCP treatment. Liangyoupeijiu (LYP9) and Nipponbare (NPBA) are rice cultivars. *P*-values of the two-way ANOVAs of 1-MCP, salt stress, and their interaction are indicated: ns, not significant; ^∗^*P* < 0.05; ^∗∗^*P* < 0.01; ^∗∗∗^*P* < 0.001. Bars with the same letter are not significantly different in the LSD test.

Biosynthesis of ethylene was enhanced in rice spikelets with increased salt stress levels. The release of ethylene in spikelets of LYP9 was higher than that in NPBA, under salt stress conditions. These findings revealed that the high concentration of ethylene in rice spikelets was considerably increased with the increased salt levels in LYP9. The same trend was shown in NPBA under CK, LS, and MS, but seedlings for NPBA at HS could not survive. The average ethylene production, from 2-years of data, in spikelets of LYP9 was significantly increased by 46, 109, and 85.8% at LS, MS, and HS, respectively, compared to the control. After usage of 1-MCP, the biosynthesis of ethylene in spikelets was decreased by 40.2% (CK), 44.3% (LS), 28% (MS), and 27.4% (HS), respectively, compared to plants without 1-MCP application (Figure 2A–D).

In the case of NPBA, the ethylene concentration was significantly increased with increasing salt levels. The increasing rate of ethylene production in spikelets, after exposure to salt stress, was 38 and 65.5% at LS and MS, respectively, compared to CK. Ethylene production in spikelets of NPBA with 1-MCP was lower at 23.9% (CK), 28.6% (LS), and 25.9% (MS) for stress levels compared to no 1-MCP treatment (Figure 2A–D). The impact of 1-MCP on reducing ethylene inhibition in spikelets of NPBA were lower than that of LYP9, under salt stress.

Ethylene biosynthesis in both LYP9 and NPBA spikelets with 1-MCP application, was significantly decreased than no 1-MCP level. 1-MCP proved its potential to inhibit ethylene action in rice under the salinity condition. The outcomes revealed that the ethylene biosynthesis in spikelets of LYP9 was more than in NPBA; this variation could be associated with the genetic variation of the cultivar response to salt stress.

#### Proline Contents in Flag Leaf

**FIGURE 3:**
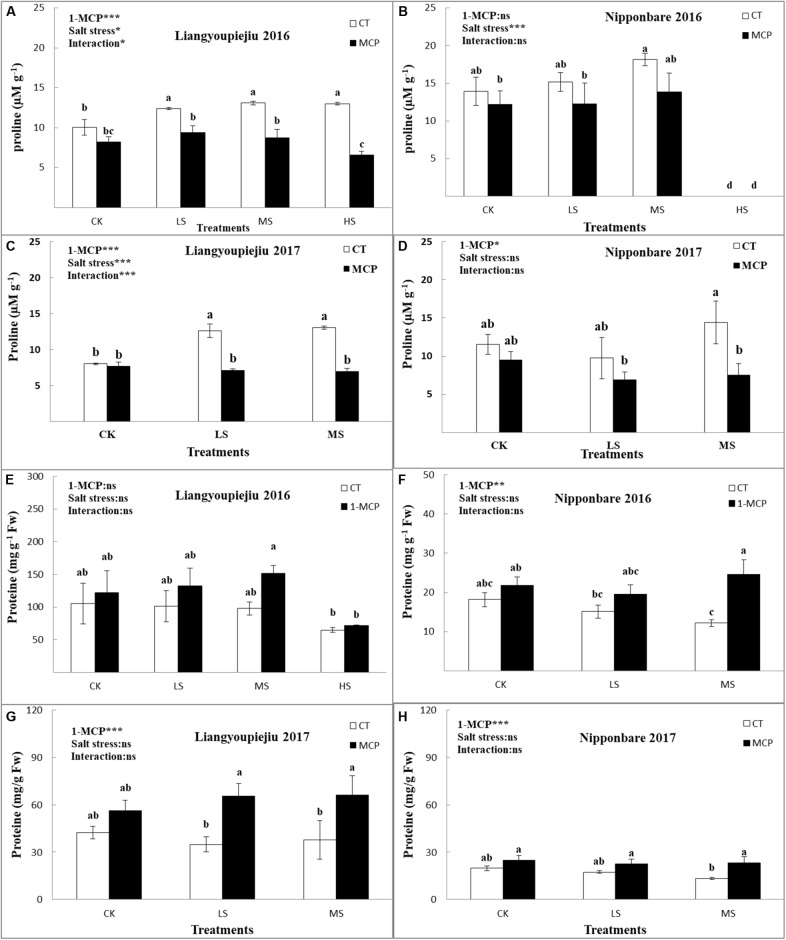
Comparison effects of 1-MCP and salt levels on proline contents **(A–D)** and protein contents **(E–H)** of rice flag leaf in 2016 and 2017. Values are denoted as mean ± SE (*n* = 4). Values followed by different letters are significantly different (*p* ≤ 0.05) according to LSD test. Where treatments are Control (CK), Low Salt Stress (LS), and Medium Salt Stress (MS), while CT = no 1-MCP treatment and 1-MCP = 1-MCP treatment. Liangyoupeijiu (LYP9) and Nipponbare (NPBA) are rice cultivars. *P*-values of the two-way ANOVAs of 1-MCP, salt stress, and their interaction are indicated: ns, not significant; ^∗^*P* < 0.05; ^∗∗^*P* < 0.01; ^∗∗∗^*P* < 0.001. Bars with the same letter are not significantly different by LSD test.

The proline contents in rice flag leaves were increased in both the LYP9 and NPBA with increasing salt levels over 2 years of experiments. In both rice cultivars, salt stress induced a boost in proline content, regardless of the tolerance to salt stress. For LYP9, the proline content in flag leaves showed significant differences between the four salt stress levels (CK, LS, MS, and HS; Figure 3A–D). The proline content in the LYP9 was 17.1–57.5% higher for LS than for CK, 23.7–62.5% higher for MS than for CK, and 9.2% higher for HS than for CK, respectively. After 1-MCP application, the proline contents were decreased by 4.3–22.4% (CK), 30.3–43.5% (LS), and 46.6–38.3% (MS), and 38.3% (HS), respectively, in comparison with LYP9 plants without 1-MCP application.

Whereas in the case of NPBA, the range of proline contents under salt stress was 8.6–15.7% (LS) and 26.4–30% (MS) higher than those for CK. When 1-MCP was applied, the proline contents were decreased by 12.8–17.4%, 19.128.9%, and 23.6–47.9%, respectively, in the CK, LS, and MS groups compared to the NPBA plants with no 1-MCP. There was a steady decrease in proline contents in the flag leaves of LYP9 under salt stress from CK to HS. After the 1-MCP application, proline contents in flag leaves decreased in both NPBA and LYP9 under salt stress. The results clearly showed that LYP9 and NPBA induced higher proline accumulation under the salinity condition. This might play a vital role for salt stress tolerance in rice cultivars. The results suggest that LYP9 and NPBA induce higher proline accumulation in a salty environment.

#### Soluble Protein in Rice Flag Leaf

I general, the soluble protein content in rice flag leaves were lower with increased salt stress levels (Figure 3E–H). Compared with CK, the soluble protein content of LYP9 decreased by 10.3, 8.3, and 38.2% in the LS, MS, and HS, respectively. Total soluble protein contents of LYP9 increased after 1-MCP application by 16.3-43% (CK), 31.4-87.4% (LS), 54.6-73.7% (MS), and 10.8% (HS), respectively, in comparison to the a comparison LYP9 group without 1-MCP. Compared with CK, the average soluble protein content of NPBA was lowered by 15.9% (LS) and 34% (MS), with increased salt stress levels. The total soluble protein content of NPBA was improved by 22.8% (CK), 20.6% (LS), and 76.5% (MS), respectively, after 1-MCP application compared to that of NPBA plants without 1-MCP application. Collectively, total soluble protein contents were higher in LYP9 than in NPBA under all salt levels. Total protein contents were higher in LYP9 than in NPBA under LS and HS salt stress (Figure 3E–H). Comparatively, LYP9 showed better results than NPBA did, due to different responses to salt stress after 1-MCP application.

#### Chlorophyll Contents (Chl *a*, *b*, Carotenoids, and Total Chlorophyll)

Total chlorophyll contents (Chl *a*, *b*, carotenoids) decreased with increased salt stress levels (Table 2). Compared with no 1-MCP application, the chlorophyll *a*, *b*, carotenoids, and total chlorophyll content increased by 7.7, 20, 14.3, and 10.6% for CK, 20, 22.5, 16.7, and 20% for LS, and 29.9, 35.6, 15.9, and 28.4% for MS, respectively in LYP9. Whereas in NPBA, the total chlorophyll contents such as chl *a*, *b*, and carotenoids increased by 11.1, 3.8, 36.5, and 13.8% for CK, 18, 13.7, 6.4, and 14% for LS, and 16.2, 44.7, 8.2, and 19.5% for MS, respectively, compared with no 1-MCP application. Total protein contents were higher in LYP9 than in NPBA in the LS and MS controls. Comparatively, LYP9 showed better results than NPBA did, because of a different response to salt levels. These results reveal that the sensitive nature of NPBA compared to LYP9. Chlorophyll contents are the basic indicators of photosynthesis activity in rice leaves. Low chlorophyll contents are one of the major causes of low growth and yield production for rice plants (Table 2).

**Table 2:** Effects of 1-MCP on chlorophyll contents (Chl *a, b*, carotenoids, and total chl contents) in 2017.

			Chlorophyll *a*	Chlorophyll *b*	Carotenoid	Total chlorophyll
Cultivars	Salt levels	PGR	mg g^-1^ Fw	mg g^-1^ Fw	mg g^-1^ Fw	mg g^-1^ Fw
**LYP9**	CK	1-MCP	2.8 ± 0.1ab	0.6 ± 0.03a	0.8 ± 0.01ab	4.2 ± 0.01a
		CT	2.6 ± 0.1b	0.5 ± 0.02ab	0.7 ± 0.03ab	3.8 ± 0.01ab
	LS	1-MCP	2.8 ± 0.2ab	0.6 ± 0.1a	0.8 ± 0.1a	4.2 ± 0.01a
		CT	2.4 ± 0.1c	0.5 ± 0.03b	0.7 ± 0.04ab	3.5 ± 0.01b
	MS	1-MCP	3.0 ± 0.1a	0.6 ± 0.02a	0.7 ± 0.1ab	4.3 ± 0.01a
		CT	2.3 ± 0.2c	0.5 ± 0.04b	0.6 ± 0.03b	3.4 ± 0.01b
	*F* Value	1-MCP	22.9^∗∗∗^	17.16^∗∗∗^	7.25^∗∗^	22.9^∗∗∗^
		Salt Stress	0.54 ns	0.47 ns	0.82 ns	0.51 ns
		Interaction	1.6 ns	0.86 ns	0.46 ns	1.44 ns
**NPBA**	CK	1-MCP	3.3 ± 0.1a	0.6 ± 0.04bc	0.9 ± 0.03a	4.7 ± 0.1a
		CT	2.97 ± 0.05b	0.5 ± 0.03bc	0.6 ± 0.04b	4.1 ± 0.1b
	LS	1-MCP	2.69 ± 0.1bc	0.6 ± 0.02b	0.7 ± 0.02b	3.9 ± 0.1b
		CT	2.28 ± 0.05de	0.5 ± 0.02bc	0.6 ± 0.05b	3.4 ± 0.05c
	MS	1-MCP	2.51 ± 0.1cd	0.7 ± 0.02a	0.7 ± 0.01b	3.86 ± 0.2b
		CT	2.16 ± 0.2e	0.5 ± 0.04c	0.6 ± 0.1b	3.23 ± 0.2c
	*F* Value	1-MCP	17.5^∗∗∗^	15.78^∗∗∗^	8.8^∗∗∗^	25.65^∗∗∗^
		Salt Stress	3.8^∗^	0.87 ns	3.9^∗^	3.5^∗^
		Interaction	28.16^∗∗∗^	5.6^∗∗∗^	2.9 ns	17.3^∗∗∗^


*The treatments are controlled (CK), low salt stress (LS), and medium salt stress (MS). LYP9, Liangyoupeijiu; NPBA, Nipponbare. *P*-values of the two-way ANOVAs of 1-MCP, salt stress, and their interaction are indicated: ns, not significant; ^∗^*P* < 0.05; ^∗∗^*P* < 0.01; ^∗∗∗^*P* < 0.001. Bars with the same letter are not significantly different in the LSD test and *n* = 6.*

### 1-MCP Modulated Antioxidant Characteristics

#### Superoxide Dismutase Activity

**FIGURE 4:**
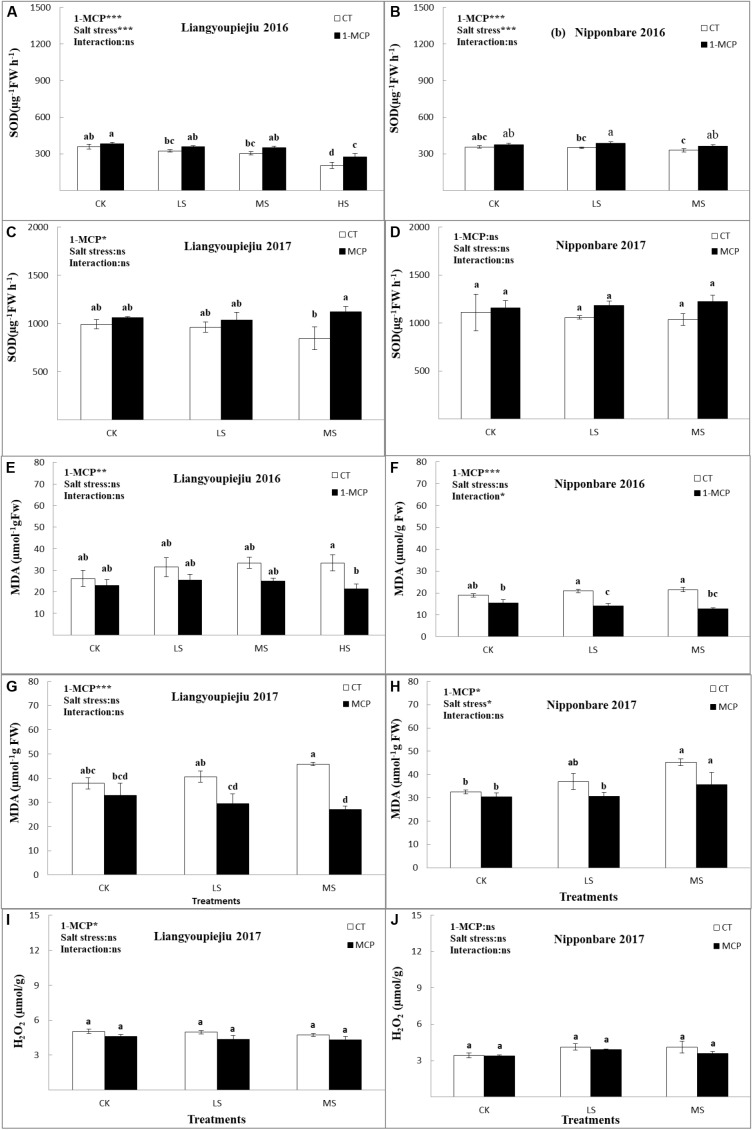
Comparison effects of 1-MCP and salt levels on SOD **(A–D)**, MDA **(E–H)**, and H_2_O_2_ **(I,J)** of rice flag leaf in 2016 and 2017. Values are denoted as mean ± SE (*n* = 4). Values followed by different letters are significantly different (*p* ≤ 0.05) according to LSD test. Where treatments are Control (CK), Low Salt Stress (LS), and Medium Salt Stress (MS), while CT = no 1-MCP treatment and 1-MCP = 1-MCP treatment. Liangyoupeijiu (LYP9) and Nipponbare (NPBA) are rice cultivars. *P*-values of the two-way ANOVAs of 1-MCP, salt stress, and their interaction are indicated: ns, not significant; ^∗^*P* < 0.05; ^∗∗^*P* < 0.01; ^∗∗∗^*P* < 0.001. Bars with the same letter are not significantly different by LSD test.

Among the enzymatic antioxidants, SOD is a key superoxide scavenger due to its enzymatic activity (Sharma et al., 2012). The results showed that SOD activity in flag leaves was decreased under all salt stress levels relative to CK in both rice cultivars. Compared to the SOD activity of CK, the SOD activity of LS decreased by 6.7%, MS decreased by 15.4%, and HS decreased by 42.7%, respectively (Figure 4A–D). 1-MCP usage improved the average SOD contents by 7.2% for CK, 8.9% for LS, 24.5% for MS, and 33.2% for HS, respectively, compared to LYP9 plants without 1-MCP application. In NPBA, the SOD activity under salt stress was 2% (LS) and 7.6% (MS) lower than CK. The average total SOD contents were improved by 4.7% (CK), 11.3% (LS), and 14.2% (MS) with 1-MCP application, compared to NPBA plants without 1-MCP application. The decreasing rate was significantly higher in LYP9 than in NPBA under salt stress. By using 1-MCP, LYP9 showed a better response than NPBA to increasing SOD contents. Decreased SOD activity under salt stress might be the most important cause of dominant membrane damage, oxidative stress, and enhanced MDA contents.

#### Malondialdehyde

Two-years of experiments revealed that increased MDA was observed with increased salt concentrations from LS to HS in LYP9, and from LS to MS in NPBA. The MDA contents in LYP9 increased by 20.2% (LS) and 27.5% (MS), respectively. MDA contents for LYP9 were lower after 1-MCP application by 13% (CK), 25.4% (LS), 33.5% (MS), and 35.6% (HS), respectively, compared to LYP9 plants without the level of 1-MCP to compare with CK. While in comparison with CK, the MDA contents in NPBA increased by 10.5% (LS) and 15.8% (MS) with increased salt stress levels. MDA contents in NPBA were lower after 1-MCP application by 13.1% (CK), 25.1% (LS), and 31.4% (MS), respectively, compared to NPBA plants without a 1-MCP level. MDA contents were higher in LYP9 than in NPBA under all salt stress levels.

Salt stress induced a boost in MDA contents in both rice cultivars, regardless of their tolerance to salt stress. For LYP9 and NPBA, the MDA contents showed a significant difference at the LS, MS, and HS levels compared to CK (Figure 4E–H). After the application of 1-MCP, MDA contents were decreased in both LYP9 and NPBA under CK, LS, MS, and HS levels. These results clearly showed that LYP9 induced higher MDA accumulation under salt stress conditions than NPBA did, which may have played a role in salt stress tolerance. However, reduced MDA contents was greater in NPBA compared to LYP9 after 1-MCP application. This variation is dependent on the genetic behavior and sensitivity of NPBA compared to the LYP9, in reaction to salt stress. These findings revealed that LYP9 might tolerate salt stress-induced oxidative damage better than NPBA.

#### Hydrogen Peroxide

Data from 2017 showed that H_2_O_2_ increased with increased salt concentrations in LYP9 and NPBA. After 1-MCP application, the H_2_O_2_ contents in LYP9 lowered by 8.5% (CK), 11.9% (LS), and 9.1% (MS), respectively, compared with no 1-MCP application. H_2_O_2_ contents in NPBA were lower after 1-MCP application by 1.5% (CK), 5.3% (LS), and 5.6% (MS), respectively, compared with no 1-MCP application (Figure 4I,J).

For LYP9 and NPBA, the H_2_O_2_ contents showed a significant difference at the LS and MS levels compared to CK (Figure 4I,J). After the application of 1-MCP, H_2_O_2_ contents were decreased in both LYP9 and NPBA under CK, LS and MS levels. However, reduced H_2_O_2_ contents was greater in LYP9 compared to NPBA, after 1-MCP usage. These results indicate that LYP9 can tolerate salt stress-induced oxidative damage better than NPBA.

### Total Aboveground Biomass

**FIGURE 5:**
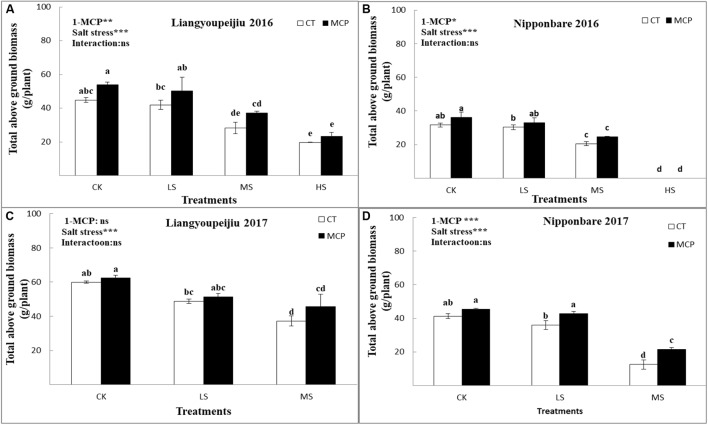
Comparison effects of 1-MCP and salt levels on total above ground biomass **(A–D)** of rice flag leaves in 2016 and 2017. Values are denoted as mean ± SE (*n* = 3). Values followed by different letters are significantly different (*p* ≤ 0.05) according to LSD test. Where treatments are Control (CK), Low Salt Stress (LS), and Medium Salt Stress (MS), while CT = no 1-MCP treatment and 1-MCP = 1-MCP treatment. Liangyoupeijiu (LYP9) and Nipponbare (NPBA) are rice cultivars. *P*-values of the two-way ANOVAs of 1-MCP, salt stress, and their interaction are indicated: ns, not significant; ^∗^*P* < 0.05; ^∗∗^*P* < 0.01; ^∗∗∗^*P* < 0.001. Bars with the same letter are not significantly different in the LSD test.

During the 2016–2017 rice growing seasons, salt stress greatly affected rice growth and total biomass production (Figure 5A–D). The total aboveground biomass in LYP9 was lowered by 12.6, 37.9, and 55.6% at the LS, MS, and HS levels, respectively, compared to CK. The total aboveground biomass for LYP9 was improved after 1-MCP application by 12.1% (CK), 12.4% (LS), 27.6% (MS), and 17% (HS), respectively, compared to without 1-MCP application.

Total above ground biomass in NPBA was also significantly lowered by 9.6% (LS) and 53.6% (MS) compared to CK. Total aboveground biomass for NPBA was higher after 1-MCP application by 11.5% (CK), 14.2% (LS), and 47.1% (MS), compared to plants without a 1-MCP level. The reduction in aboveground biomass was noticeably higher in NPBA, perhaps due to its salt sensitivity feature (Figure 5A–D). In comparison, 1-MCP significantly improved the total aboveground biomass in both rice cultivars. This might be because of a high response to 1-MCP in NPBA (a salt sensitive cultivar).

The effect of 1-MCP on increasing the total aboveground biomass was more obvious for LYP9 than for NPBA under salt stress conditions. These variations in total aboveground biomass may be due to the osmotic, ionic, and hormonal variations caused by salt stress. 1-MCP treatment performed better regulating results in LYP9 under adverse salt stress conditions. LYP9 showed susceptibility against the HS level, whereas NPBA could not survive at the HS level. These results suggested that NPBA is a highly sensitive rice cultivar.

### Grain Yield

**FIGURE 6:**
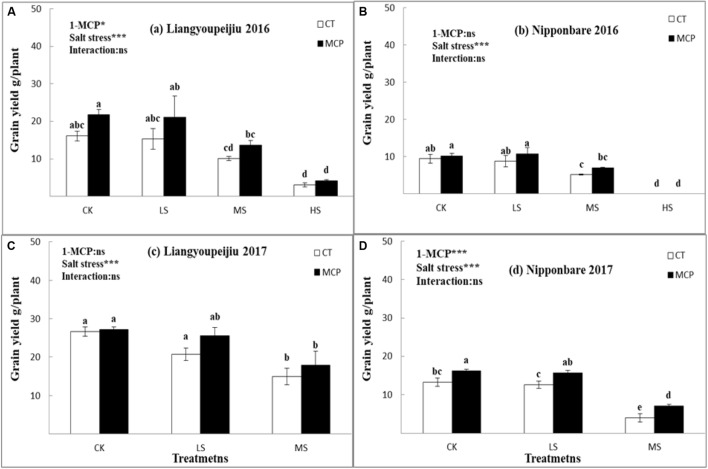
Comparison effects of 1-MCP and salt levels on yield per plant **(A–D)** in 2016 and 2017. Values are denoted as mean ± SE (*n* = 4). Values followed by different letters are significantly different (*p* ≤ 0.05) according to LSD test. Where treatments are Control (CK), Low Salt Stress (LS), and Medium Salt Stress (MS), while CT = no 1-MCP treatment and 1-MCP = 1-MCP treatment. Liangyoupeijiu (LYP9) and Nipponbare (NPBA) are rice cultivars. *P*-values of the two-way ANOVAs of 1-MCP, salt stress, and their interaction are indicated: ns, not significant; ^∗^*P* < 0.05; ^∗∗∗^*P* < 0.001. Bars with the same letter are not significantly different in the LSD test.

Salt stress commonly reduces rice grain yield as salt levels increase. During the 2016–2017 experiments, the average grain yield per plant for LYP9 decreased by 14.3% (LS) and 41.6% (MS), and 85.5% (HS), compared with that of CK. In the NPBA cultivar, with an increased salt level, rice grain yield reduced by 4.7 and 52.3% under LS and MS, compared with no salt stress (CK), respectively. The application of 1-MCP increased the grain yield of both LYP9 and NPBA, with various salt levels (Figure 6A–D). In the LYP9, the mean value of rice grain yield was 19.4% (CK), 30.3% (LS), 26.4% (MS), and 34.5% (HS) higher with 1-MCP application than without 1-MCP application, compared to similar salt stress levels.

The rice grain yield per plant after application of 1-MCP in NPBA was 15.1% (CK), 24% (LS), and 55.4% (MS) higher than that without 1-MCP application. The rice seedlings died under heavy salt stress levels. Two-year results suggested that salt stress could adversely affect rice grain yield, while 1-MCP application could positively improve the yield.

## Discussion

High salt concentrations in soil enhanced the ethylene biosynthesis and affected the homeostasis of rice growth features and physiology (Sajid et al., 2018). The use of 1-MCP as an ethylene action inhibitor, to manage oxidative stress in rice plants, showed a close relationship to physiological, biochemical, antioxidants responses, and total above ground biomass, along with grain yield per plant to salt stress in rice. However, this is dependent on the level of ethylene receptors, the sites of the tissues, and synthesis. Salt stress might play an important role in the reaction to ethylene and will help us understand the effects of 1-MCP (Kevany et al., 2007). Many researchers also reported that receptor specification isoforms have special roles in specific physiological reactions to stress (Binder et al., 2006; Kevany et al., 2007). Increased salt stress imposed oxidative, osmotic, enzymatic, and hormonal imbalances on plant growth and might cause plant death (Cotsaftis et al., 2011), so a slight increase in salt levels in a plant cell resulted in adverse effects on the rice lifecycle. Salt stress affects numerous physiological, agronomical, and biochemical processes, i.e., superoxide dismutase (SOD), malondialdehyde (MDA), H_2_O_2_, proline, and chlorophyll contents, e.g., chl *a*, *b*, carotenoids. Moreover, it also affects soluble protein accumulation and growth characteristics such as aboveground biomass production and yield (Mostofa et al., 2015; Parihar et al., 2015). 1-MCP is a unique ethylene action inhibitor; 1-MCP has a low molecular weight and shows ten times more affinity to inhibit ethylene than other inhibitors because of its special α-cyclodextrin (α-CD), and is environmentally friendly (In et al., 2013; Kawakami et al., 2010). Moreover, 1-MCP also contributes to rice plant growth regulating functions (Wang et al., 2012; Zhang et al., 2015).

The outcome of this 2-year experiment suggests that the exogenous usage of 1-MCP results in encouraging effects on physiological and agronomic characteristics and shows significant effects on the homeostasis of biochemical characteristics, especially SOD, MDA, H_2_O_2_, proline, soluble proteins and chlorophyll contents. The less effective results of 1-MCP on NPBA than LYP9 may be because of the highly sensitive nature of NPBA. The LYP9 cultivar showed a high susceptibility against heavy salt stress levels (HS).

Salt stress negatively affected the physiological characteristics (P_n_ and SPAD values) of rice under increased salt concentrations. In this experiment, these parameters were reduced by salt stress in both LYP9 and NPBA. The application of 1-MCP significantly improved the P_n_ and SPAD values under CK, LS, MS, and HS in LYP9. However, the NPBA plant died at the HS level. This might be due to the involvement of 1-MCP in improving the physiological and molecular mechanisms by the positive 1-Aminocyclopropane-1-carboxylic acid synthase (ACS), 1-aminocyclopropane-1-carboxylate oxidase (ACO), cell-wall-transforming enzymatic mechanisms, and the expression of *GhCel1* in the abscission region of the plant leaf, which hinders the breaking of the tissue of leaves due to ethylene action inhibition (Mishra et al., 2008; Zhang et al., 2014). 1-MCP improves the photosystem II quantum efficiency, increases membrane integrity, and delays leaf senescence, ultimately enhancing the physiological characteristics of the plant (Loka and Derrick, 2013; Chen et al., 2015; shown in Figure 1A–H).

Ethylene is a stress plant hormone that when enhanced under unfavorable environments (salt stress), leads to leaf senescence and early maturity of fruits, finally causing yield reduction (Quinet et al., 2010; Loka and Derrick, 2013). From methionine the S-AdeMet (SAM), the primary ethylene precursor changes into 1-Aminocyclopropane-1-carboxylic acid (ACC) using 1-Aminocyclopropane-1-carboxylic acid synthase (ACC synthase) and finally produces ethylene biosynthesis in response to the synthase oxidase (ACC oxidase) under a salt stress environment Pandey et al., 2000; (Lin et al., 2009). 1-Aminocyclopropane-1-carboxylic acid (ACC) has passive effects on ethylene production in the plant under stress conditions (Rudus et al., 2013; Zhang et al., 2014). High ethylene biosynthesis in rice spikelets is among the key grain yield-decreasing factors in salt-affected soil environments (Sajid et al., 2018). High ethylene production affects the physiology of the rice plant and passively disturbs assimilation of starch (Mohapatra and Panigrahi, 2011). It also affects the accumulation of starch contents in rice grains through the ethylene receptor *ETR2* responsible monosaccharide transport gene (Wuriyanghan et al., 2009).

Therefore, in order to enhance rice grain yield, management approaches to ethylene action hang-ups are needed in amalgamation with improvements in genetic engineering under salt affected conditions (Yang et al., 2007). The use of an ethylene action binder improved the development of rice spikelets and plant dry biomass (Zhang et al., 2014, 2015; Sajid et al., 2018). Numerous studies revealed that ethylene inhibitors are a useful tool in coordinating the development of rice spikelets, especially inferior spikelets of japonica and indicia rice cultivars (Wang et al., 2012; Zhang et al., 2014, 2015; Sajid et al., 2018).

In this research experiment, the use of 1-MCP as an ethylene action inhibitor meaningfully stopped the ethylene-induced mechanism in spikelets under unfavorable salt affected environment levels such as CK, LS, MS, and HS in both LYP9 and NPBA rice cultivars (Figure 2A–D). This inhibition of ethylene action in rice spikelets is due to the unique behavior of 1-MCP as an ethylene action inhibitor. 1-MCP has an affinity to fix the ethylene receptors sites irretrievably. Consequently, signaling translation and transduction reactions are not motivated, and it chunks the protein synthesis and enzymatic action mechanisms through high ethylene production (Arigita et al., 2003; Su and Finlayson, 2012) and plants grow normally after this phenomenon.

Agronomic traits, i.e., aboveground biomass production (Figure 5A–D). This reduction is due to an osmotic, ionic, and hormonal imbalance especially in ethylene effects in rice plants, which vary in rice growth stages, as observed by Zeng and Shannon (2000); Shibli et al. (2007), Zhang et al. (2014), and Sajid et al. (2018). The decreased length of roots and shoots under abiotic stress ultimately decreased Pn mechanism, which resulted in less production of plant biomass (Amirjani, 2011; Sajid et al., 2018). Zhang et al. (2014) and Sajid et al. (2018) found similar results in the effects of 1-MCP on growth parameters and the development of rice spikelets under normal field and salt stress conditions. Based on total above ground biomass production at the maturity stage, we can classify both cultivars, since LYP9 was susceptible to the HS levels, and NPBA was highly sensitive at the HS level.

Grain yield per plant reduced with increased salt stress levels (Figure 6A–D). The reduced grain yield per plant under salt stress levels was caused by the reduction of soluble sugar (starch) content transformation in rice spikelets and the starch synthetase activity decrease during grain development (Abdullah et al., 2001). These findings revealed that 1-MCP plays a remarkable role in the regulation of the flag leaf functions by improving the Pn and SPAD value, resulting in an improvement in rice spikelets under salt stress. In the present study, 1-MCP increased the rice grain yield of LYP9 and NPBA cultivars under controlled and salt stress (Figure 6A–D).

Under salt stress conditions, membrane lipid peroxidation (MDA) is an indicator of cell membrane leakage and rupturing (Katsuhara et al., 2005). Malondialdehyde was increased in rice with increased salt stress levels. Hossain et al. (2013) and Kaur et al. (2016) reported a similar trend of increasing MDA contents in rice leaf tissues under salt stress. The steady escalation of MDA observed in this study is a clear indication that salt stress significantly affected oxidants and caused a rapid osmotic adjustment that released oxidative stress. This trend signifies that an increase in MDA is useful to protect plant mechanisms from salt stress in rice cultivars LYP9 and NPBA (Figure 4E–H). These results were also observed in other cereal crops such as wheat, and our outcomes are supported by Goudarzi and Pakniyat (2009); Srivashtav et al. (2015), and Kaur et al. (2016).

Proline proved to be an osmoprotectant as well as an osmoregulatory through osmotic effects due to abiotic stresses (salt stress) and enabled the plant to sustain development (Srivashtav et al., 2015). Higher proline synthesis in transgenic rice causes an improvement in salt tolerance (Srivashtav et al., 2015). Proline concentration plays a crucial role in protecting the cellular structures, especially the protein structure, against oxidative damage through scavenging the free radicals produced by abiotic stressors such as salt stress, chilling, and drought (Silva et al., 2013). Proline contents increased with increasing NaCl concentrations in plant leaf tissues. In this study, the increase in proline is useful to protect plant mechanisms against salt stress in rice cultivars LYP9 and NPBA, though the NPBA cultivar could not survive in high salt stress (Figure 3A–D). Ahmed et al. (2013) and Islam et al. (2015a) found the same results in rice and barley plants under salt and drought stress. Proline induced by salt stress might be an acclamatory response to improve salt tolerance in rice cultivars (Parihar et al., 2015). High proline in LYP9 under all salt stress levels showed its susceptibility to salt stress (Figure 3A–D).

In this study, the protein contents decreased with increasing NaCl concentration in plant leaf tissues of both rice cultivars LYP9 and NPBA (Figure 3E–H). Protein contents were higher in LYP9 than in NPBA under salt stress. This variation shows that a higher accumulation of protein in LYP9 flag leaves than in NPBA, protecting the cell from salt stress. Salt stress causes an imbalance in ions which involve in the synthesis of soluble protein and photosynthesis and cause degradation of chlorophyll contents (Di Martino et al., 2003). The reduction of protein contents in flag leaves might be due to an Na^+^ and K^+^ imbalance in plant tissues. Decreasing K^+^ passively affects the protein synthesis and cause the destruction of plant growth features (Chen et al., 2007). 1-MCP improved the protein contents in both rice cultivars under salt stress conditions (Figure 3E–H).

SOD (Superoxide Dismutase) is a key superoxide (O^-2^) scavenger because of its enzymatic action. In this study, increased salt concentration decreased SOD contents in rice flag leaves. A similar study was done by Benitez et al. (2013). These results suggest that the reduction in SOD activity, due to salt stress, could be a major factor of significant membrane damage and increased MDA content under oxidative stress (Figure 4A–D). The decrease in SOD activity in rice leaves follows the “O_2_” accretion in leaf cells, and consequently, they block related enzymatic and non-enzymatic activities (Sarwar et al., 2003; Jajic et al., 2015). Similar results were found in cotton crops under stress when 1-MCP was used (Chen et al., 2015).

H_2_O_2_ is a basic element of oxidative metabolism. It is a product of peroxisomal and chloroplastic oxidative reactions, and it is an active oxygen species (Del Rio et al., 1992). The increase of ROS seems to occur as a response to abiotic stresses such as salt stress. H_2_O_2_ might perform a vital role in the salt injury mechanism (Singha and Choudhuri, 1999). H_2_O_2_ accumulation, caused by salinity, has been studied in many plants, e.g., Vigna catjang, rice, and pea plants (Hernandez et al., 1993). H_2_O_2_ was important in regulating salt injury in our rice leaf system. In this study, there was no significant effect under salt stress and 1-MCP application. The activity of GO, the enzymes which catalyze the synthesis of H_2_O_2_ in the peroxisome, remained unchanged in control leaves. These results are very important for the homeostatic mechanism in ROS and antioxidants (Figure 4I,J).

Total chlorophyll contents (chlorophyll *a*, *b*, carotenoids) reduced in concentration, possibly due to the inhibition effect of the accumulation of various ions caused by salts stress, in the production of the various chlorophyll contents (Ali et al., 2004). In this study chlorophyll contents decreased with increased salt stress. Our results are supported by Adnan and Erkan (2016). This decrease in the Chlorophyll content in rice flag leaves might be because of the increase in variability pigments protein complexity and the activity of the Chls enzyme (Beinsan et al., 2009) Table 2.

The mitigating effects of the 1-MCP application on SOD, MDA, H_2_O_2_, proline, protein, and chlorophyll contents, i.e., chl *a*, *b*, and carotenoids, and total chlorophyll contents, in rice flag leaves under various salt levels, could be caused by its role in the rice plant as a signaling molecule. 1-MCP inhibits ethylene action and triggers plant defense responses and modulate homeostasis for cellular redox, including biochemical mechanisms, ion balance, and responses to oxidative stress (Sharma et al., 2012).

## Conclusion

Salt stress significantly decreased agronomical, physiological, and biochemical characteristics of rice plants, as well as increased the ethylene production in spikelets. Application of 1-MCP resulted in favorable responses to salt stress, and 1-MCP proved to be an effective ethylene inhibitor. 1-MCP could not only significantly inhibited ethylene action in rice spikelets but, also improved physiological characteristics, such as P_n_ and SPAD values under increased salt levels in both rice cultivars. 1-MCP also improved agronomical and biochemical characteristics such as SOD, soluble protein, chlorophyll content, and decreased MDA, H_2_O_2_ contents, and proline in flag leaves of rice. The homeostasis of these characteristics, due to 1-MCP application, resulted in improvements in aboveground biomass, and grain yield for rice plants under various salt stress levels. The usage of 1-MCP proved to be one of the most important strategies for enhancing the rice plant’s performance in response to salt stress. The results provided a strong base for the modulation of physiological, agronomical, and biochemical characteristics of rice under salt stress, through the use of 1-MCP. In future, our focus is to further study the effects of 1-MCP on genetic and molecular responses to salt stress in the rice plant.

## Author Contributions

SH, JZ, and QJ conceived and designed the study and conducted the experiments. JH, XC, CZ, ZB, and LZ contributed the analytical tools. SH and CZ analyzed the data. SH and JZ wrote the manuscript. MK revised the manuscript. The manuscript has been revised and approved by all authors.

## Conflict of Interest Statement

The authors declare that the research was conducted in the absence of any commercial or financial relationships that could be construed as a potential conflict of interest.
